# Ultrasonic evaluation of plantar fascia in patients with osteoarthritis of the knee

**DOI:** 10.1097/MD.0000000000030613

**Published:** 2022-09-23

**Authors:** Zongjie Liu, Xin Sui, Ran Hui, Ying Zhao, Hua Li, Xiaodan Huang

**Affiliations:** a Department of Ultrasound Medicine, The Thrid Hospital of Hebei Medical University, Shijiazhuang, Hebei Province, People’s Republic of China; b Department of Orthopectics, The Thrid Hospital of Hebei Medical University, Shijiazhuang, Hebei Province, People’s Republic of China.

**Keywords:** knee osteoarthritis, Plantar fascia, ultrasound

## Abstract

To study the changes of plantar fascia in patients with knee osteoarthritis. Collect knee arthritis surgery patients and according to the length of the course is divided into long-course and short-course group, collection of healthy volunteers as control group at the same time, basic information such as age, height, weight, and body mass index (BMI) were recorded; the application of Philips and Siemens ultrasonic diagnostic instrument, a foot plantar fascia in patients with knee osteoarthritis in ultrasonic scanning, measuring the thickness of the heel of plantar fascia, observe its sonographic manifestation; age, BMI, and plantar fascia thickness were compared between groups. The plantar fascia thickness of the normal control group was 0.30 ± 0.06 cm on the left side and 0.30 ± 0.05 cm on the right side. The plantar fascia thickness of the long-course group was 0.44 ± 0.10 cm on the left side and 0.42 ± 0.10 cm on the right side. The plantar fascia thickness of the group with short course of disease was 0.37 ± 0.06 cm on the left side and 0.34 ± 0.7 cm on the right side. Multivariable analysis of variance was used to compare the thickness of plantar fascia in the long-course group, the short-course group, and the control group, *P* < .05; there were statistical differences among the 3 groups. Multivariate analysis of variance was used to compare the general data of the long-course group, the short-course group, and the control group. Age: the long-course group was compared with the short-course group and the control group, *P* < .05; short-course group compared with control group, *P* > .05. BMI: compared with long-course group and short-course group, *P* < .05; long course of disease group compared with short course of disease group, *P* > .05. BMI was statistically different between the case group and the control group. Plantar fascia was thickened in patients with knee osteoarthritis, and the thickening of plantar fascia was related to BMI. The thickening of plantar fascia was uneven, and the degree of thickening was related to the course of disease. At the same time, the sonogram of plantar fascia was less echogenic than that of normal controls.

## 1. Introduction

Knee osteoarthritis (OA) is a chronic bone and joint disease with a high incidence in China. It mainly invades bone, articular cartilage, and synovial tissue. Severe OA will cause patients knee pain, accompanied by knee varus and valgus deformity or flexion contracture deformity. The abnormal function and morphological changes of knee joint caused by OA directly affect the changes of lower limb mechanical force line, thus causing gait changes^[1,2]^ Some scholars have found that during the development of OA, the alignment of the whole limb including the ankle joint is changed, thus causing ankle arthritis.^[3]^ Plantar fascia is a part of plantar fascia which can sense the orientation and the muscle contraction state inside the foot, so as to coordinate the functional state of plantar muscles, nerves, and other structures during walking, and also buffer the vibration and impact of plantar during walking and movement. It is an important supporting structure for maintaining stability of the foot.^[4]^ Plantar fasciitis is a common problem that 1 in 10 people will experience in their lifetime. Risk factors include increased body mass index (BMI), limited ankle dorsiflexion, and standing for prolonged periods of time.^[5]^ Then does plantar fascia change in patients with knee OA? It has not been studied at present. Therefore, this paper mainly studies whether and how plantar fascia changes in patients with knee OA.

High-frequency ultrasound is more and more widely used in musculoskeletal examination. It has the following advantages: high resolution in the examination of motor system, especially superficial tissues; safe, noninvasive, nonradioactive, applicable to a wide rang of people; Able to dynamically observe the condition of the healthy side and the affected side by real-time imaging; observe the changes of blood flow and the presence or absence of inflammation; and musculoskeletal minimally invasive interventional therapy can be performed under the guidance of ultrasound.^[6]^

Normal plantar fascia ultrasound showed a thin band with strong echo, and the average thickness of the heel was about 3.0 ± 0.5 mm.^[7]^ In this paper, the thickness and ultrasonic manifestation of plantar fascia at heel were observed in patients with OA.

## 2. Methods

### 2.1. Subjects

The severe OA inpatients with a medical history of >5 years and requiring knee replacement surgery were diagnosed as the long-course group. The inpatients with confirmed severe OA with a medical history of <5 years and requiring knee replacement surgery were diagnosed as the short-course group. At the same time, healthy volunteers were collected as control group.

Inclusion criteria for patients with OA: (1) in accordance with OA clinical manifestations: patients with knee joint pain, limited function with flexion and extension, (2) in accordance with severe OA X-ray manifestations: joint space narrowing obviously even disappear or accompanied by osteophyte formation, sclerotic bone, cystic degeneration changes, etc. (3) No clinical manifestations of fasciitis include heel pain, which is obvious when getting up in the morning and relieved after activity.^[5]^ (4) Check and confirm the patients’ side knee joint fit the surgical indications of total knee replacement surgery.

Inclusion criteria for the normal population: patients with no related bone and joint diseases, no plantar fasciitis diseases, and no diseases affecting lower limb activities, such as foot deformity, diabetic foot, and neurological diseases, can be included in the control group of the normal population by inquiring about the medical history of bone and joint diseases and physical examination.

### 2.2. Instruments and methods

Philips and Siemens ultrasonic diagnostic instrument are performed, with probe frequency of 5 to 12 MHz and 3 to 9 MHz. The client lay flat on the examination bed with both feet hanging over the edge of the bed, exposing the plantar plantar fascia. The same senior sonographer scanned the plantar fascia. First, the proximal plantar fascia was displayed by 2-dimensional ultrasound scanning, and the internal echo, boundary, and the relationship between the upper and lower edges of the plantar fascia and the surrounding tissues were observed. Meanwhile, measuring the thickness of the plantar fascia at 1 to 2 cm from the insertion point of calcaneal nodular. The probe was positioned longitudinally and measure the thickness of the thickest plantar fascia transversely. Then the blood flow of plantar fascia was observed by color Doppler ultrasonography. All the ultrasound operators are professional sonographers who have worked for >5 years and skillful operation. The protocol was approved by Medical Ethics Committee of Hebei Province.

### 2.3. Statistical methods

SPSS 13.0 was used for data analysis, and measurement data were expressed as “*X ± S*”. Multivariate analysis of variance was used for comparison between groups, and *P* < .05 was considered statistically significant.

## 3. Results

### 3.1. General information

The long-course group: a total of 99 patients with severe OA were treated from April 2019 to April 2020, including 15 males and 84 females with an average age of 65.39 ± 7.62 years, the average height of 1.61 ± 0.07m, the average weight of 73.11 ± 11.41 kg, and the average BMI was 28.28 ± 4.14 kg/m^2^.

The short-course group: a total of 40 patients with severe OA were treated from April 2019 to April 2020, including 15 males and 25 females with an average age of 60.08 ± 6.96 years, the average height of 1.64 ± 0.08) m, the average weight of 72.82 ± 10.57g, and the average BMI was 26.95 ± 3.37 kg/m^2^.

The control group: a total of 66 healthy volunteers were collected as control group, including 26 males and 40 females, with an average age of 58.55 ± 14.05 years. The average height was 1.64 ± 0.08 m, the average weight was 66.31 ± 10.33 kg, and the average BMI as (24.55 ± 3.29).

### 3.2. Sonographic manifestations of plantar fascia

**Figure 1.**
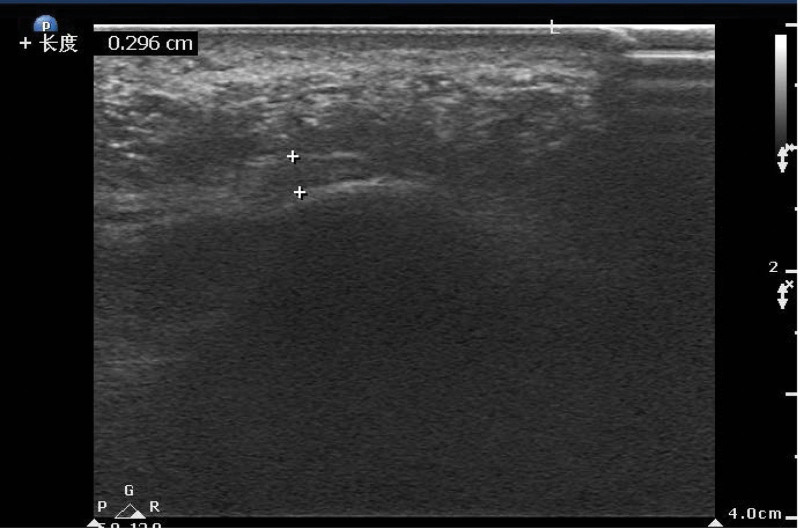
Normal plantar fascia in 2-dimensional ultrasonic longitudinal section was thin band with medium-high echo, and uniform parallel line with high echo, about 0.30 cm in longitudinal section.

In the normal group, the plantar fascia in 2-dimensional ultrasonic longitudinal section was thin band with medium-high echo, and uniform parallel line with high echo (Fig. 1). The upper and lower edges of plantar fascia and surrounding tissues had clear boundary, and the plantar fascia in transverse section was crescent-shaped structure with thickening in the middle and gradually thinning on both sides, and the internal echo was uniform.

**Figure 2.**
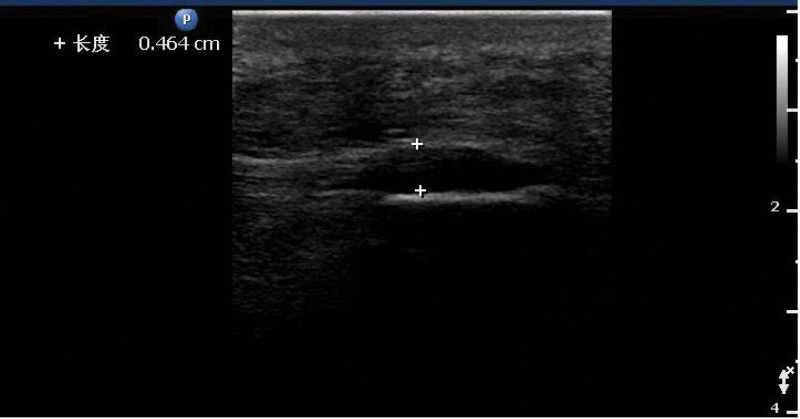
In patients with knee osteoarthritis, plantar fascia was thickener and echo decreased compared with normal control group (Fig. 1).

**Figure 3. and 4.**
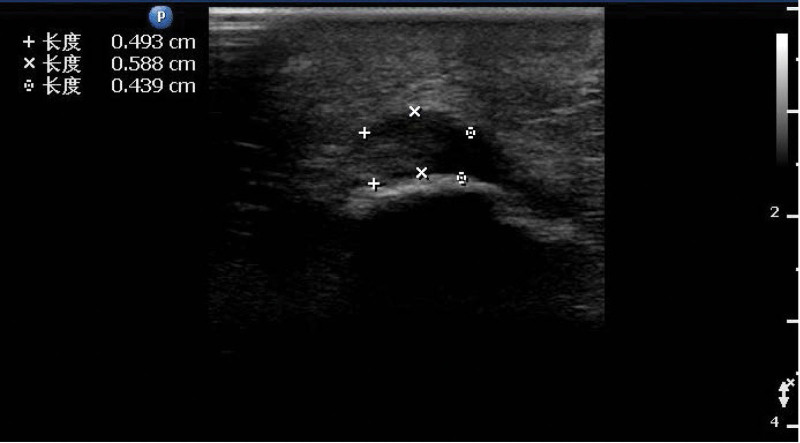
Transverse section of plantar fascia in patients with knee osteoarthritis, with uneven thickening of plantar fascia and uneven internal echo.

**Figure 4.**
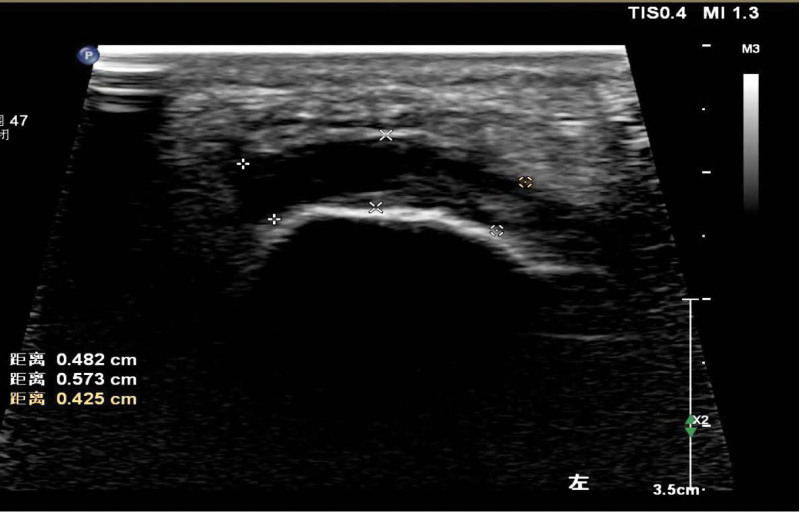
Transverse section of plantar fascia in patients with knee osteoarthritis, with uneven thickening of plantar fascia and uneven internal echo.

Patients with knee arthritis plantar fascia echo significantly decreased (Fig. 2), the internal echo was uneven, the parallel line hyperecho was blurred or disappeared, and the boundary between the upper and lower edges of fascia and surrounding tissues was not clear. Measuring the transverse section thickness of the plantar fascia thickening, the thickest area is located in the middle, visibly inside and outside uneven thickening either (Figs. 3 and 4), color Doppler blood flow signal not seen obviously increase. The plantar fascia was thickened to varying degrees in the long- and short-course groups.

### 3.3. Plantar fascia thickness

The plantar fascia thickness of the normal control group was 0.30 ± 0.06 cm on the left side and 0.30 ± 0.05 cm on the right side. The thickest one is about 0.45 cm. The plantar fascia thickness of the long-course group was 0.44 ± 0.10 cm on the left side and 0.42 ± 0.10 cm on the right side. The thickest one is about 0.73 cm. The plantar fascia thickness was >0.50 cm in 50 patients (25.25%), including 21 on the left side and 29 on the right side. The plantar fascia thickness of the group with short course of disease was 0.37 ± 0.06 cm on the left side and 0.34 ± 0.7 cm on the right side, and the thickest was about 0.56 cm. There were 4 feet with plantar fascia thickness >0.50 cm, accounting for about 5%, including 2 feet on the left and 2 feet on the right. Multivariable analysis of variance was used to compare the thickness of plantar fascia in the long-course group, the short-course group, and the control group, *P* < .05; there were statistical differences among the 3 groups.

Multivariate analysis of variance was used to compare the general data of the long-course group, the short-course group and the control group. Age: the long-course group was compared with the short-course group and the control group, *P* < .05, the long-course group was older than the short-course group, and the long course group was older than the control group; short-course group compared with control group, *P* > .05, there was no significant difference in age between the short-course group and the control group. BMI: the control group compared with long-course group and short-course group, *P* < .05, BMI of the long-course group and the short-course group was higher than the control group; long-course group compared with short-course group, *P* > .05, BMI of the long-course group and the short-course group had no statistical difference. There was statistical difference in BMI between the case group and the control group, suggesting that the thickening of plantar fascia was related to BMI.

In conclusion, at the same age, BMI increased in the case group compared with the control group, and so did plantar fascia thickness. BMI of the long-course group was the same as that of the short-course group, and the thickness of plantar fascia in the long-course group was thicker than that in the short-course group, suggesting that the thickness of plantar fascia in patients with degenerative osteoarthritis of the knee joint was related to the course of the disease, and the thickness of plantar fascia was thicker in the long course of the disease.

## 4. Discussion

OA is a common middle aged and elderly people with bone and joint diseases; the incidence is high in China, with sclerosis of subchondral bone hyperplasia, joint, trabecular bone enlargement, increased local osteoclast, bone destruction^[8]^ as the main pathological characteristics, clinical symptoms mainly for knee pain, deformation, and activity obstacle; the X-ray is characterized by joint gap narrowed or even disappear, subchondral densification, sclerosis, and cystic degeneration. Epidemiological investigation found that OA was related to gender, age, weight, occupational nature, and living environment.^[9]^ Through meta-analysis, Tie et al^[10]^ found that the total prevalence of OA in middle aged and elderly people >40 years old in China was 17.0%, and the prevalence of OA in females was higher than that in males. The cases collected in this study basically conform to the epidemiological distribution, with female cases significantly more than male cases.

Plantar fascia is a kind of aponeurotic structure that runs along the plantar lengthways, has strong quality and tension, and supports the biometrical function of the bottom of the foot. It is a thick fibrous connective tissue located in the subcutaneous fibrous layer of the bottom of the foot. It originates from the medial tubercle of the calcaneus and is inserted into the phalanges distally. The plantar fascia is attached to the metatarsal surface of the calcaneus below the calcaneal tubercle, and the fibers of the plantar fascia and the Achilles tendon are interwoven to attach to the posterior calcaneal metatarsal.^[11]^ Plantar fascia protects and supports some important plantar anatomical structures, such as muscles, bones, nerves, and blood vessels, and can maintain the normal shape of the arch of the foot, guarantee the extension and dorsiflexion of the plantar toe joint, and limit the excessive plantar flexion and dorsiflexion of the foot. Anatomical studies of feet show that the thickness of the medial, middle, and lateral plantar fascia is different.^[12]^ This study observed the visible heel of plantar fascia ultrasonic transverse normal control group for middle thickness thinning in both sides, the moon or curved, but cases have taken place in its shape is irregular change, and in the case group, the duration is > 5 years than the course of <5 years but not more obvious thickening, BMI, there was no significant statistical difference in 2 cases. It is suggested that the thickening of plantar fascia in patients with OA is related to the course of disease. Studies^[13]^ have shown that thickness of plantar fascia >5 mm on ultrasound or magnetic resonance imaging indicates plantar fascia disease. In this study, irregular thickening of plantar fascia was observed in patients with OA, and the thickness of plantar fascia in some patients was >5 mm, accounting for 25.25% and up to 7.3 mm, which may indicate that OA can also be complicated by plantar fascia disease. Since none of the patients in this study had obvious clinical symptoms such as heel pain, it may be suggested that plantar fasciitis caused by the development of OA is asymptomatic fasciitis. There were no obvious clinical symptoms such as heel pain, and no significant increase in blood flow signals of the thickened plantar fascia during ultrasound examination, which was different from the clinical image manifestations of plantar fasciitis, or the thickened plantar fascia in patients with OA was only a compensatory change, which played a role in enhancing shock absorption and stability maintenance. Further clinical study is needed.

Plantar fasciitis is a chronic degenerative disease that causes pain in the medial heel of plantar fascia. Patients with plantar fasciitis have obvious pain after the first steps of the day or after standing for a long time.^[14,15]^ During physical examination, palpation along the medial calcaneus of the plantar can produce painful symptoms. Long-term repetitive overload pulling can lead to chronic inflammation of fibrous tissue, hyperplasia, fibrosis, degenerative changes, and eventually plantar fasciitis.^[16]^ Some studies^[17–19]^ believed that plantar fasciitis was a kind of mechanical injury. Ultrasound and stress analysis were used to analyze the thickness and stress of plantar fasciitis, and it was confirmed that plantar fasciitis was thickened. Pain is related to stress in the plantar area. Dai et al^[20]^ showed that the biomechanical characteristics of plantar in plantar fasciitis patients showed that the contact area of the medial part of the posterior foot with the most obvious pain was smaller, and the plantar pressure was more inclined to the forefoot and lateral part of the foot. Zhang et al^[21]^ showed that the plantar pressure of patients with osteoarthritis also changed, and the lateral plantar pressure of patients decreased, resulting in uneven plantar stress and pathological gait. This study showed that there was a statistical difference in BMI between the case group and the control group, suggesting that the thickening of plantar fascia was related to BMI, and that the thickening of plantar fascia was related to weight overload. In the case group, the metatarsal fascia thickening was more obvious in patients with a course of disease >5 years than in patients with a course of disease <5 years, and there was no significant difference in BMI between the 2 groups, which may indicate that the metatarsal fascia thickening in patients with OA is related to the course of disease. Therefore, we consider that the change of plantar fascia in patients with knee arthritis may be related to the long-term overload pull of plantar fascia caused by the change of physical force line after knee arthritis. However, long-term follow-up is still needed to observe how the thickened plantar fascia changes after adjustment of the force line after knee replacement.

In conclusion, plantar fascia in patients with OA was thickened inhomogeneously, and the degrsee of thickening was related to the course of disease. Meanwhile, the sonogram of plantar fascia was less echogenic than that of normal controls.

## Acknowledgments

We thank technician Fang Qinmao their technical assistance and for providing valuable suggestions.

## Author contributions

**Conceptualization:** Liu Zongjie,Sui Xin

**Data curation:** Zhao Ying,Liu Zongjie

**Formal analysis:** Liu Zongjie

**Funding acquisition:**Liu Zongjie,Hui Ran

**Investigation:** Liu Zongjie,Hui Ran,Huang Xiaodan,Li Hua,Zhao Ying

**Resources**:Huang Xiaodan

**Project administration**: Liu Zongjie,Sui Xin,Hui Ran

**Supervision**:Liu Zongjie,Sui Xin

**Writing—original draft:** Liu Zongjie

**Writing—review & editing:** Liu Zongjie, Sui Xin
